# EEG glasses for real-time brain electrical activity monitoring

**DOI:** 10.1038/s41598-025-29893-4

**Published:** 2025-11-29

**Authors:** Renato Zanetti, Amir Aminifar, David Atienza

**Affiliations:** 1 Department of Electronics and Biomedical Engineering, Federal Center For Technological Education of Minas Gerais (CEFET-MG), Belo Horizonte, 30510-000 Brazil; 2 Institute of Electrical and Micro Engineering, Swiss Federal Institute of Technology Lausanne (EPFL), Lausanne, 1015 Switzerland; 3 Department of Electrical and Information Technology, Lund University, Lund, 22100 Sweden

**Keywords:** Engineering, Health care, Neurology, Neuroscience

## Abstract

Wearable devices are becoming a cornerstone for personalized and long-term health monitoring, enabling early intervention and data-driven medical decisions. In this work, we present *e-Glass*, a state-of-the-art smart wearable device that enables unobtrusive real-time electroencephalography (EEG) monitoring. Our evaluation shows that *e-Glass* adheres to the established international guidelines for clinical EEG recordings. Moreover, the acquired data presents a Pearson’s correlation of 0.93 relative to recordings obtained from the Biopac research-grade EEG system . The proposed EEG acquisition device concept is evaluated in two application domains: epileptic seizure detection and cognitive workload monitoring (CWM). First, we present a lightweight edge machine-learning scheme, designed specifically for *e-Glass*, achieving overall sensitivity of 64% (100% sensitivity in 11 out of 24 subjects) and 2.35 false-alarms per day, when tested on 982.9 hours of EEG data from the CHB-MIT dataset. Similarly, an CWM strategy with *e-Glass* reaches an accuracy of 74.5% on unseen data. These results demonstrate that *e-Glass* is capable of unobtrusive and real-time subject monitoring in outpatient conditions, not only in epileptic seizure detection but also in monitoring the subject’s cognitive state.

## Introduction

The United Nations projects that one in six people will be over 65 years old by 2050, most of them living in low- and middle-income countries^1^. As the population ages, the burden on national health systems is expected to increase, requiring smart solutions to help bend the cost curve of the current hospital-centered treatment structure. Among existing candidates, wearable devices are expected to contribute by providing continuous and personalized clinical data to enable early intervention and data-driven medical decisions^2^. By leveraging edge Machine Learning (edge-ML) and Internet-of-Things (IoT), wearables are the cornerstone to monitor, detect, and predict health conditions in real time^3^, e.g., in epileptic seizure detection^4–8^.

Epilepsy is a brain disorder characterized by recurrent seizures that shows a prevalence of 4 to 8 per 1000 population, placing it as the fourth most common neurological disorder worldwide^9^. In this use case, wearable-based epilepsy monitoring is considered an essential tool for patient management, providing real-time seizure tracking and alerts to patients and caregivers, which could prevent, for example, Sudden Unexpected Death in Epilepsy (SUDEP)^10^. Currently, People With Epilepsy (PWE) can choose from a few validated solutions on the market: *Epi-Care*, *Empatica*, and *Nightwatch*^11^. These devices are validated for generalized tonic–clonic seizures (GTCS), which are characterized by strong movements, reaching reasonable performance metrics using only accelerometers and/or a few peripheral biosignals. However, GTCS represent less than 15% of all seizures faced by PWE^12^. Therefore, we require the use of electroencephalography (EEG) to tackle other seizure types, as brain activity monitoring is the golden standard to diagnose and monitor PWE^12^.

Existing commercially-available wireless EEG acquisition systems are, in general, cumbersome and stigmatizing, rendering them unacceptable to PWE^13^. As such, the development of wearable EEG faces several challenges. First, PWE requires inconspicuous form factors to avoid discrimination^14^. Second, they also require devices presenting low false-alarm rates (FAR) and high sensitivity to consider its adoption and long-term engagement^11^. The state-of-the-art (SoA) algorithms for subject monitoring rely on many EEG channels (i.e., a minimum of 18 channels), which are not available on wearable EEG devices^7,15,16^. To compensate for the reduced number of EEG channels of wearable EEG, we would require specialized and complex ML algorithms. However, the target devices are built on resource-constrained platforms (e.g., reduced memory, processing capacity, and battery size), to ensure portability, wearability/comfort, and unobtrusiveness.

To tackle the ML-based detection challenge, in our previous work, we pioneered the usage of only four EEG electrodes over the frontotemporal lobes to detect epileptic seizures that would fit a smart glasses form factor^4,17,18^. The eyeglasses form factor is also preferred for the AttentivU platform^19^, which targets attention and engagement feedback using one bipolar EEG channel over TP9 and TP10 scalp positions^20^. The work in^21^ presents a smart glasses for wearable healthcare and Human-Machine Interfaces (HMIs) combining EEG and electrooculography (EOG). More recently, the work in^22^ introduced GAPses, a smart glasses also integrating EEG and EOG data processing on a RISC-V processor (GAP9, Greenwaves Technologies) to tackle Steady-State Visual Evoked Potential, Motor Movement classification, and EEG-based biometrics (BrainMetrics). Although these platforms are versatile, they do not directly target epileptic seizure detection.

**Fig. 1:**
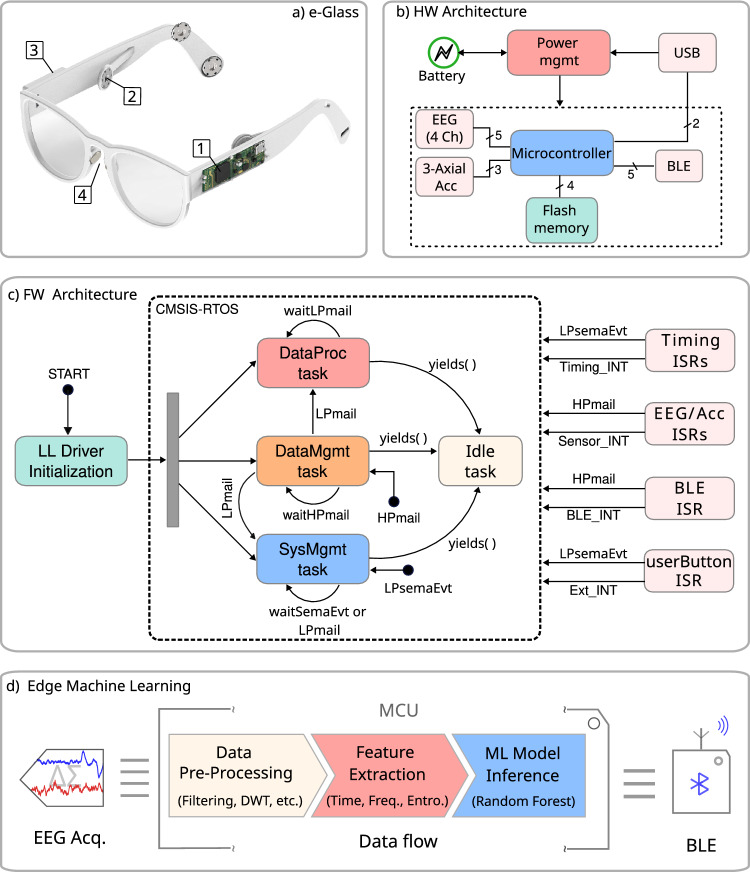
(**a**) Proposed *e-Glass* system (parts: 1- main board, 2- Electrode snap connector, 3- battery casing, 4- bias electrodes); (**b**) hardware block diagram for *e-Glass*’ main board; (**c**) firmware state diagram, in which the main state displays four main threads used by *e-Glass*’ RTOS; (**d**) edge-ML scheme representing the data flow and processing within *e-Glass*.

In this work, we present *e-Glass*, a state-of-the-art smart wearable system for real-time EEG monitoring targeting epilepsy. The *e-Glass* design concept, presented in Figure 1a, is meant for achieving an inconspicuous and unobtrusive wearable EEG by design, which are two essential characteristics to address the social stigma and discrimination problems. Moreover, e-Glass can deliver high performance on seizure detection for PWE using only a few EEG electrodes thanks to a tailored ML-based data processing framework optimized for resource-scarce devices ^4,7,17^. Finally, *e-Glass* real-time EEG processing can also be used for cognitive workload (CW) monitoring (CWM)^23^. Our main contributions are as follows:

- we present a state-of-the-art wearable platform for unobtrusive EEG acquisition and processing in real-time, namely *e-Glass*, targeting personalized monitoring of PWE. Moreover, we present a lightweight edge-ML scheme tailored for *e-Glass*, which has limited resources (compute, memory, and energy).
- we perform the electrical characterization of *e-Glass*’ hardware and assess the system’s EEG acquisition capability. *e-Glass* prototype presents as low as 0.16 $\mu$ $V_{RMS}$ of input-referred noise (IRN) and 18.41 noise-free bits (NFB), adhering to the guidelines for digital recording of clinical EEG of the International Federation of Clinical Neurophysiology (IFCN)^24^. Moreover, *e-Glass* acquired signals reaches up to 0.93 of average Pearson’s correlation with the data from a research-grade EEG equipment, demonstrating similar band-power and alpha-wave synchronization during experimental tests.
- we tackle the seizure detection problem, proposing a data processing framework tailored for ML-based EEG processing in resource-scarce devices as *e-Glass*. Testing this framework on all available data of the CHB-MIT dataset^15^ (982.9 hours of data), it reaches an overall sensitivity of 64% (100% sensitivity in 11 out of 24 subjects) and 2.35 false-alarms per day, considering only two bipolar EEG channels (i.e., F7T7 and F8T8). *e-Glass* can run up to 28.5 hours of operation on a single battery charge (225 $mA\cdot h$ battery) when executing the proposed seizure detection scheme.
- we explore a solution for real-time CWM on wearable devices composed of an ML design methodology and a data processing strategy, both validated on an experimental dataset. The proposed solution reaches an accuracy of 74.5% and a 74.0% geometric mean of sensitivity and specificity on unseen data.

In the following sections, first, we present the *e-Glass* system, describing hardware, firmware, and the ML-based data processing scheme adopted for its applications. Next, we extensively evaluate *e-Glass*, in terms of electrical characterization and EEG acquisition capabilities, as well as its performance in the context of epileptic seizure detection and cognitive workload monitoring application domains. Finally, we detail the methodology adopted for evaluating the device and conclude this work.

## *e-Glass*

*e-Glass* is a state-of-the-art wearable device that builds on the conventional eyeglasses design, a widely-accepted form-factor of wearable technology, to allow unobtrusive real-time brain electrical activity monitoring (i.e., EEG). By integrating this capability into an everyday accessory, we aim to mitigate the social stigma and discrimination often associated with using bulky commercial EEG equipment^13,14^. Figure 1 depicts *e-Glass* system, its hardware and firmware main blocks, followed by the ML scheme used to process EEG onboard in real time. In the following, we describe each system component in detail.

### *e-Glass* system and hardware architecture

The *e-Glass* hardware is embedded within the temples of the eyeglasses to maintain the appearance of a conventional wearable device. Figure 1 presents (a) the *e-Glass* system along with its detailed hardware architecture in the form of (b) a system block diagram. Four main parts are marked within Figure 1a: (1) the device’s main electronic board for data acquisition, processing, and communication; (2) a snap connector for the EEG electrodes; (3) the casing for the rechargeable ion-lithium battery; (4) EEG bias electrodes, to be discussed later. The *e-Glass* hardware is structured into four main subsystems: signal acquisition, data processing unit, communication interface, and onboard memory. Besides, we employ energy-efficient components, prioritizing subsystems capable of standalone operation and generating interrupt signals to the microcontroller whenever an intervention is needed.

*e-Glass* is designed based on ADS1299 front-end^25^ to provide four EEG channels using a referential electrode montage. We adopt the scalp positions *F*7, *T*7, *F*8, and *T*8, according to the 10–20 International Electrode System^20^, which were investigate previously for seizure detection^4^. The reference electrode is placed over the left mastoid bone. Additionally, the system incorporates an active bias electrode to drive a body point with a voltage potential within the system’s power rails and boost the common-mode rejection ratio (CMRR). The bias electrode is required to increase the systems CMRR, which can provide at least 13 dB gain^26^.

Concerning electrodes, *e-Glass* is designed to use soft-dry electrodes. This type of electrodes offer advantages in outpatient settings, requiring no conductive gel and minimal skin preparation. However, their use introduces challenges related to increased susceptibility to noise due to impedance mismatches. To address this issue, voltage followers are placed close to each electrode, implementing the concept of active electrodes within *e-Glass*^27^. This approach significantly increases the circuit’s input impedance as seen by each electrode, thereby reducing the voltage divider effect between the skin-electrode interface and the circuit input impedance. Furthermore, active electrodes enable on-site current amplification, enhancing signal strength and lowering cabling impedance, effectively mitigating noise caused by capacitive coupling.

### *e-Glass* firmware architecture

The *e-Glass* firmware is built on the ARM CMSIS (Cortex Microcontroller Software Interface Standard) Real-Time Operating System (CMSIS-RTOS)^28^, utilizing STMicroelectronics low-level (LL) drivers and hardware abstraction layers (HAL)^29^. The adoption of an RTOS offers several advantages, including (1) deterministic behavior, ensuring predictable execution times and resource access during critical tasks; (2) an event-driven architecture that optimizes system responsiveness; (3) sophisticated scheduling algorithms that enable efficient task parallelism; and (4) robust, ready-to-use application programming interfaces (APIs) for memory management and self-monitoring, facilitating debugging and system reliability.

Figure 1c illustrates a simplified representation of the *e-Glass* firmware operation. As previously mentioned, an event-driven architecture was adopted to enhance energy efficiency, leveraging queues, mail queues, and semaphores to synchronize data transfers and task execution. Additionally, microcontroller external events are managed through interrupt service routines (ISRs), as shown on the right side of the super-state.

The four primary tasks within the *e-Glass* RTOS structure are encapsulated within the super-state labelled *CMSIS-RTOS*. Additional tasks were implemented to manage data communication via USB and Flash memory access. The main tasks are as follows:

- *SysMgnt* – responsible for monitoring and controlling the basic functionalities of *e-Glass*, including push buttons, battery management, and system status;
- *DataMgmt* – manages all data-related events, including Bluetooth Low Energy (BLE) communication and signal acquisition (e.g., EEG and accelerometer data);
- *DataProc* – executes real-time data processing routines for user monitoring;
- *Idle* – a default RTOS task that uses the CPU when no other task is ready for execution.

Shared resources among tasks are synchronized using the *HPmail* and *LPmail* mail queues and the *LPsemaphEvt* semaphore. For instance, *DataMgmt* remains blocked while awaiting a *HPmail* message from an ISR (e.g., BLE or EEG front-end). These messages are stored in a FIFO queue, ensuring timely data processing. To prevent data loss or communication failures, *DataMgmt* is assigned the highest priority. For real-time processing, *DataMgmt* generates a *LPmail* for *DataProc*, then yields execution to trigger a context switch. It also sends *LPmail* messages to *SysMgnt* for BLE-based system adjustments or user requests. When all tasks are blocked, the *Idle* task runs. To reduce power consumption, the RTOS Idle hook enables tickless operation, suspending periodic tick interrupts until an event or task transition occurs. Upon resumption, the RTOS tick count is adjusted using the real-time clock count.

To minimize average current consumption, *e-Glass* leverages the low-power modes of the ARM Cortex-M4. The strategy prioritizes keeping the microcontroller in power-save mode, activating tickless operation whenever possible^30^. The system operates at its maximum core clock (i.e., 80 MHz) to efficiently execute tasks. When a task enters the blocked state, another task may run, if available; otherwise, the *Idle* task triggers low-power mode. Additionally, unused peripherals are clock-gated, while active peripherals alternate between running and low-power states to further reduce consumption.

The available ISRs, shown in light pink, include timing resources, external chip interrupts, and the user button interface. *e-Glass* utilises two timing sources: one for the CMSIS, based on a microcontroller hardware timer, and an RTC for the system-wide timestamping. External interrupts handle EEG front-end signals and accelerometer data availability. The signal acquisition subsystem stores all generated data in a circular buffer. When a data batch is ready, a *HPmail* is sent to the *DataMgmt* task. Similarly, the BLE ISR feeds a FIFO buffer and triggers a *HPmail* upon receiving a message.

### *e-Glass* edge-ML

The hardware restrictions inherent in resource-constrained wearable devices increase the challenge of monitoring subjects in real-time and in outpatient settings, on a long-term basis. Nevertheless, current microcontroller architectures include advanced features such as single instruction multiple data (SIMD) and accelerators, larger RAM and Flash memory, and ultra-low-power modes, enabling the execution of tailored digital signal processing algorithms. Such characteristics foster the adoption of the edge computing paradigm and embedded ML to tackle the battery lifetime problem, thanks to the much lower power consumption of computation over communication^23,31,32^.

Leveraging the hardware and firmware features of *e-Glass*, we implemented real-time processing algorithms to support the basic application flow described in Figure 1c. We utilise the CMSIS-DSP library^28^, optimised for ARM microcontrollers, for algorithm implementation. However, to address CMSIS-DSP’s functional limitations, we supplement it with a cross-compiled GNU Scientific Library (GSL) for data processing. The basic building blocks include:

- Data pre-processing: (1) sample-wise average removal to tackle high amplitude baseline wander and artifacts, to reduce signal overshooting when (2) band-pass filtering the data using an infinite impulse response (IIR) filter; (2) a lightweight artifact removal algorithm tailored for microcontroller-based systems with just a few EEG channels^23^; (3) GSL’s Discrete wavelet transform (DWT) to enhance time-frequency EEG features.
- Feature extraction: classical features in EEG-based applications on time domain – line length, mean amplitude, mean, variance, standard deviation, and Hjorth parameters^33,34^, EEG power distribution per EEG band^15^, and EEG complexity estimations based on Shannon, Tsallis, Rényi, Sample, and Permutation entropy as defined in^35^.
- ML model inference: Random Forest (RF) based inference, leveraging the power of ensemble models to achieve high accuracy with low data overfitting^36^.

Each edge ML block can be used independently or combined to achieve a custom application according to the accuracy requirements and the energy budget. An *e-Glass* application is expected to employ at least the filtering module, feature extraction algorithms, and an ML model to infer the subject’s condition regarding epilepsy monitoring or CW.

## Results and discussion

In this section, first, we detail the results for the *e-Glass* electrical characterization in terms of input-referred noise level and other important parameters with regard to a guideline for digital recording of clinical EEG of the IFCN^24^. Second, we present the experimental results of *e-Glass* EEG acquisition in comparison with Biopac BN-EEG2 (Biopac Systems, Inc.), a commercial research-grade equipment. Lastly, we discuss the main results for two forecasting applications – seizure detection and cognitive workload monitoring (CWM).

### *e-Glass*’ hardware electrical characterization

Excessive circuit noise can degrade the performance of analog-to-digital converters (ADC), particularly in low signal-to-noise ratio (SNR) applications like EEG acquisition. Key ADC parameters are critical for achieving low-noise data acquisition. The ADS1299 datasheet provides figures for input-referred noise (IRN), dynamic range (DR), noise-free bits (NFB), and effective number of bits (ENOB) across different sampling rates and input gains. Moreover, the IFCN has proposed a set of guidelines for digital recording of clinical EEG, which indicates: 1) minimum sampling frequency (Fs) of 200 samples/s; 2) minimum resolution of 0.5 $\mu$V; 3) input impedance of 100 M$\Omega$ or more; 4) CMRR of at least 110 dB for each channel measured at the amplifier input; 5) IRN of less than 1.5 $\mu$V peak-to-peak at any frequency from 0.5–100 Hz^24^.

**Table 1:** Comparison to IFCN guidelines. IRN for [0.5, 100] Hz bandwidth. CMRR measure for a 50 Hz input sinusoidal signal of 100 $mV_P$. Measurements executed with for PGA=24 V/V and $F_S=250~Hz$.

	Fs (Hz)	Resolution ($$\mu$$V/bit)	Impedance ($$M\Omega$$)	CMRR (dB)	$$IRN$$ ($$\mu$$$$V_P$$)
IFCN^24^guidelines	$\ge 200$	$\le 0.5$	$\ge 100$	$\ge 110^1$	$\le 1.5$
*e-Glass* system	250	0.023	$>1000$	$110^2$	1.07

^1^This value shall be at the amplifier input.1 This value shall be at the amplifier input.

^2^Value given by ADS1299 datasheet2 Value given by ADS1299 datasheet.

Table 1 compares IFCN guidelines and *e-Glass* electrical characteristics obtained by the IRN measurement and CMRR determination. The use of the ADS1299 front-end, designed explicitly for EEG acquisition, allows *e-Glass* system to outperform the IFCN guidelines. The minimum sampling frequency of ADS1299 is already greater than the required value while providing the lowest acquisition noise. Regarding input impedance, the input voltage follower has a maximum input bias current of 1 pA. As the input resistance is measured by the input-current change over input-voltage change, *e-Glass*’ input impedance is expected to be superior to 1 G$\Omega$. Moreover, the oversampling and filtering mechanisms available in $\delta$ $\sigma$ analog-to-digital converters (ADC), as in ADS1299, explain the substantially inferior IRN of *e-Glass* at a specific frequency (i.e., 50 Hz in the assessed data) concerning IFCN. Finally, ADS1299 guarantee at least 110 dB of CMRR at the amplifier imput, as required by the IFCN standard. However, we also assessed *e-Glass*’ open-loop CMRR, measuring it at the EEG electrode snap connector, which achieves 89.1 dB already considering the degradation caused by input protection and filtering. Nonetheless, this value shall increase dynamically by at least 13 dB boosted by the BIAS circuit^26^. Therefore, *e-Glass* would present at least 102 dB of CMRR, which is considered satisfactory as the target frequency band is 30 Hz or less.

**Table 2:** *e-Glass*’ static noise measurement and performance parameters. Noise figures for PGA=24 V/V, $F_S=250~Hz$, and [0.5, 65] Hz bandwidth.

	IRN		NFB	ENOB	DR
	($$\mu$$$$V_{RMS}$$)	($$\mu$$$$V_P$$)	(bits)	(bits)	(dB)
*e-Glass*	0.16	1.07	18.41	19.64	118.24
ADS1299’s Datasheet	0.14	0.98	18.54	19.85	119.5

Finally, excessive circuit noise can degrade the performance of ADC. Table 2 shows the results for IRN measurement along with other key ADC performance parameters as dynamic range (DR), noise-free bits (NFB), and effective number of bits (ENOB). In its datasheet, ADS1299 presents IRN values obtained as an average of the measurement of various demonstration boards but disconsider external input circuitry, which adds noise (e.g., Johnson–Nyquist noise). The *e-Glass* measurement is taken from the electrode snap connector input, in a more conservative approach. Thus, the slightest higher IRN obtained indicates the quality o *e-Glass* hardware design.

### *e-Glass*’ EEG acquisition assessment

**Fig. 2:**
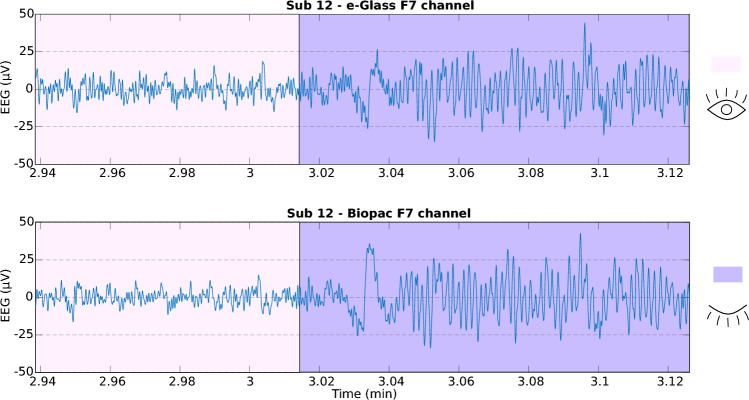
Alpha band synchronization during closed eyes, observed on *e-Glass* and BN-EEG2 for their electrodes on F7.

**Fig. 3:**
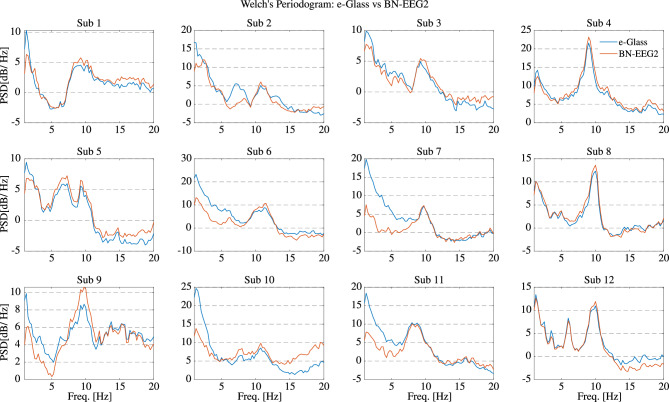
Welch’s Periodogram for the EEG acquired on the F7 electrode the subject remained with eyes closed. *e-Glass* signal’s PSD is shown in blue.

**Fig. 4:**
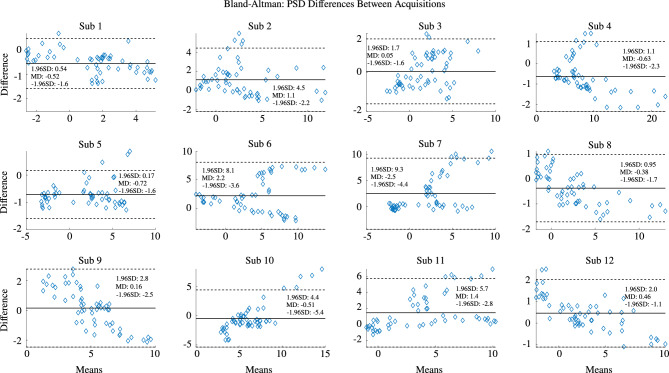
Bland-Altman plot: *e-Glass*’ minus Biopac BN-EEG2’s PSD values versus their means, for the data acquired on the F7 electrode. In the figure, MD refers to mean difference and SD to standard deviation.

In addition to bench tests, we assess *e-Glass* capacity to acquire brain activity based on a comparative analysis against Biopac’s BN-EEG2, a commercial research equipment^37–39^. First, Fig. 2 presents short EEG example acquired from Sub. 12 simultaneously by *e-Glass* and BN-EEG2. A distinct alpha wave (i.e, EEG synchronization around 10 Hz) is visible following eye closure, which is an indicative of brain activity acquisition^40^. Second, we obtain the power spectrum density (PSD) from spontaneous EEG recorded during the eyes-closed part of Task 1 (Session 1) of the experimental setup. As presented in Figure 3, alpha-band (i.e. [8–12]Hz) energy synchronization can be observed in most of the subjects (e.g., Sub 4, Sub 8, Sub 12). Moreover, a visual assessment shows a strong correlation between *e-Glass* and BN-EEG2 data’s PSD. In some cases, *e-Glass* signals exhibit greater energy in the lower frequency range (0.5–3 Hz), likely due to electrode-skin impedance mismatches common on systems using dry electrodes^41^. The Bland-Altaman scatterplot^42^ in Fig. 4 allows for assessing the agreement between the two methods of EEG acquisition, focusing on the PSD differences. We observe a small mean bias between measurements for all subjects and small differences in PSD (the majority of data points are within the 95% confidence interval).

Finally, Table 3 presents Pearson’s correlation coefficients obtained after applying the Dynamic Time Warping (DTW) on 1 minute of EEG data to account for the difference in sampling frequency between systems. *e-Glass* signals showed a high correlation with Biopac’s for most subjects, reaching an average correlation of 0.93 on the F7 electrode during eyes open task. The high correlation obtained indicates that *e-Glass* system can produce an equivalent measurement of a subject brain activity to a commercial research-grade device. Notably, the lower correlation observed for the F7 signal during the eyes-closed state in Subject 10 is attributed to a high-amplitude blinking artifact, as confirmed by visual inspection of the data.

**Table 3:** Pearson’s correlation beteween *e-Glass* and Biopac acquired signals.

Subjects	CH	01	02	03	04	05	06	07	08	09	10	11	12	AVG±STD
Eyes Open	F7	0.96	0.94	0.95	0.96	0.98	0.96	0.87	0.94	0.96	0.79	0.81	0.95	0.93±0.06
	T7	0.90	0.68	0.90	0.96	0.77	0.92	0.87	0.90	0.93	0.92	0.79	0.94	0.88±0.08
Eyes Closed	F7	0.94	0.93	0.93	0.98	0.97	0.84	0.91	0.96	0.95	0.30	0.78	0.97	0.88±0.18
	T7	0.89	0.90	0.89	0.97	0.93	0.70	0.79	0.92	0.93	0.91	0.72	0.96	0.88±0.09

**Table 4:** Seizure detection: performance metrics obtained using only *e-Glass* EEG channels versus 18 channels available among all subjects (full-cap). We include the number of seizures of each subject (# Seiz) and how many of them were used in the ML testing. The FAR metric is given per day.

Subjects		01	02	03	04	05	06	07	08	09	10	11	12	13	14	15	16	17	18	19	20	21	22	23	24	Avg
# Seiz		7	3	7	4	5	10	3	5	4	7	3	27	12	8	20	10	3	6	3	8	4	3	7	16	185
# Test		5	2	3	3	4	7	2	3	3	6	2	16	11	4	19	9	1	5	2	7	3	2	4	9	132
*e-Glass*	Sens	1.00	0.50	1.00	0.33	1.00	0.00	0.50	1.00	1.00	1.00	1.00	0.94	0.27	0.25	0.68	0.00	0.00	0.40	1.00	0.43	0.00	1.00	1.00	1.00	0.64
	Prec	0.38	1.00	0.75	0.50	0.50	0.00	1.00	0.50	1.00	1.00	1.00	0.63	1.00	1.00	0.76	0.00	0.00	1.00	1.00	0.60	0.00	0.67	0.80	0.53	0.65
	F1	0.56	0.67	0.86	0.40	0.67	0.00	0.67	0.67	1.00	1.00	1.00	0.75	0.43	0.40	0.72	0.00	0.00	0.57	1.00	0.50	0.00	0.80	0.89	0.69	0.59
	FAR	5.41	0.00	0.73	0.17	2.91	0.00	0.00	4.80	0.00	0.00	0.00	14.4	0.00	0.00	2.83	0.00	6.00	0.00	0.00	2.73	1.74	1.72	1.15	11.8	2.35
Full-Cap	Sens	1.00	1.00	1.00	0.67	1.00	0.29	0.50	1.00	1.00	1.00	1.00	0.94	0.27	1.00	0.84	0.00	1.00	0.40	1.00	1.00	0.33	1.00	1.00	1.00	0.80
	Prec	0.63	0.50	0.75	0.67	0.80	0.67	1.00	0.43	1.00	1.00	1.00	0.68	0.75	0.80	0.80	0.00	0.50	1.00	1.00	0.70	1.00	1.00	0.67	0.69	0.75
	F1	0.77	0.67	0.86	0.67	0.89	0.40	0.67	0.60	1.00	1.00	1.00	0.79	0.40	0.89	0.82	0.00	0.67	0.57	1.00	0.82	0.50	1.00	0.80	0.82	0.73
	FAR	2.03	2.40	0.73	0.17	0.73	0.41	0.00	6.40	0.00	0.00	0.00	11.2	1.50	1.14	2.83	0.00	1.50	0.00	0.00	4.10	0.00	0.00	2.30	5.90	1.80

**Table 5:** CWM: Cross-validation and testing performance after feature selection (18 features total for e-Glass and 33 for the full-cap configuration) and hyperparameter optimization to reduce the model size (200 trees for e-Glass and 400 for the full-cap). We use 19 EEG channels on the full-cap evaluation.

System Config.	CV (%)		Testing (%)				Model Size (KB)
	Acc	Gmean	Sens	Spec	Acc	Gmean	
e-Glass	$75.8 \pm 4.7$	$75.1\pm 5.0$	82.9	66.1	74.5	74.0	113.5
Full-Cap	$82.0 \pm 5.2$	$81.3\pm 5.8$	73.3	87.7	80.5	80.2	645.7

### Application results

Table 4 summarizes the performance metrics for seizure detection obtained using Times-Slice Cross-Validation (TSCV) scheme. Using data from 18 EEG channels (i.e., full-cap), our approach achieves an average sensitivity of 80%, correctly identifying 99 out of 132 tested seizures. However, as discussed before, such a solution is certainly not acceptable to PWE, due to the stigma associated with the EEG caps/hats. When limited to *e-Glass* electrodes, sensitivity decreases to 64%, as expected, given that some subjects (e.g., CHB17 and CHB21) lack ictal activity in the frontotemporal lobes. Nevertheless, the proposed method achieves 100% sensitivity in eleven subjects, demonstrating its potential for PWE monitoring based on wearable devices.

Moreover, achieved FAR of 2.35/day and 1.81/day, for the e-Glass and the full-cap solution, respectively, are close to the target of one false alarm per day even considering the low EEG-channel count of *e-Glass*^43^. These results are on par with the work in^44^, a similar SoA work that reached average sensitivity of 65.27% combining seizure and artifact detection to reduce FAR, but showing up to 24 false alarms per day, compared to *e-Glass* with only 2.35 false alarms per day. Even when compared with a work which employs convolution networks to detect seizures using a minimum of 18 EEG channels, *e-Glass*’s average F1 and sensitivity (i.e., 59% and 64%, respectively) is on par with the F1 of 59% and sensitivity of 58.3% presented in^16^, but achieving much lower FAR than this work’s 0.5/h (i.e., 12/day).

In addition to seizure detection, we also evaluate e-glass in the context of CWM. Similarly to seizure detection results, our proposed approach for CWM using four monopolar EEG channels shows only a 6.2% lower geometric mean (Gmean) of sensitivity and specificity in the test set opposed to the full-cap solution (Table 5). Moreover, the we propose an RF model optimization that reduces Flash memory requirements by 5.7x with respect to the full-cap one. Similar differences are observed for the other performance scores. Although the full-cap provides slightly higher scores, such a solution would not fit the user’s desire for an inconspicuous wearable EEG device^13,14^. Finally, our results are on par with the SoA. For example, the study in^45^ achieved an accuracy of 77% for the same dataset, but using only the full-cap solution, and the work in^46^ achieves accuracy of 80.2% with the need of using four peripheral biosignals, both works targeting cumbersome systems. Therefore, our proposed solution based on *e-Glass* achieves satisfactory results, on an low EEG-channel’s count device, toward enabling awareness of the operators’ cognitive states on HMI.

Finally, our proposed edge-ML methodology has a very low latency, taking 26.45 ms to process each seizure detection inference and 56.4 ms on the CWM one. This leaves the CPU idle for up to 97% of the time, which allows us to reduce the *e-Glass* average current consumption to approximately 7.8 mA (ADS1299 always on draws around 4.6 mA), leading to up to 28.5 hours of operation on a single battery charge (225 $mA\cdot h$ battery). Regarding RAM memory, the CLF algorithm demands up to 4774 bytes on the seizure detection application, while the CWM one uses up to 8256 bytes due to extra signal storage for the artifact removal algorithm.

## Methods

**Fig. 5:**
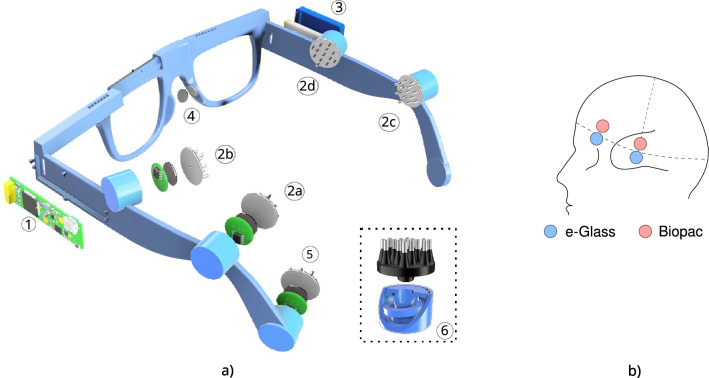
e-Glass Prototype: (**a**) *e-Glass*’ prototype with a piston-like electrode housing and the *SoftPulse* soft-dry electrodes from *Datwyler* (Switzerland Inc); (**b**) Electrode position for experimental data acquisition.

To validate the *e-Glass* system, we developed a prototype based on an adjustable 3D design, to be able to fit to various head sizes among the subjects (Fig. 5a). This prototype incorporates a piston mechanism for electrode housing with a built-in spring to increase electrode-skin pressure and dampen small vibrations to improve EEG signal to noise ratio. In the following paragraphs, we present the methodology adopted to perform the electrical characterization of *e-Glass*’ hardware and the experimental setup to assess the system’s EEG acquisition capability. The latter has been executed in comparison with a commercial equipment from *Biopac Inc*, for which EEG has been acquired with both systems at the same time and close electrode positions as displayed in Fig. 5b. Lastly, we also present two foreseeing applications for *e-Glass* device and describe the ML strategy we adopt to evaluate them.

### *e-Glass* prototype: hardware design and validation

Figure 5a presents *e-Glass* 3D design including: (1) *e-Glass*’ main electronic board for data acquisition, processing, and communication; (2) four EEG channels, including active electrodes and the proposed piston-like electrode housing; (3) ion-lithium battery casing; (4) bias electrodes; (5) reference electrodes; (6) the piston-like electrode housing and the *SoftPulse* soft-dry electrodes from *Dätwyler Holding Inc.*.

All components in the *e-Glass* electronic board are off-the-shelf, industry-grade, ensuring suitability for medical applications. For instance, the ADS1299 is leveraged on the prototype to reach a compact EEG front-end offering up to 22 *nV*/*bit* resolution and <1 $\mu \hbox {V}_{RMS}$ IRN. Moreover, while simultaneously acquiring data from four channels, ADS1299 consumes only 22mW (4.6mA) in active mode. Second, for data processing, the *e-Glass* prototype employs an STM32L476 microcontroller, featuring an ultra-low-power ARM 32-bit Cortex-M4 architecture with 1 MB Flash and 128 KB RAM. Running at up to 80 MHz, it achieves 1.25 DMIPS/MHz (Dhrystone 2.1 benchmark). The microcontroller supports nine power modes and clock-gating to optimize energy efficiency, consuming just 1.4 $\upmu$A in *Stop2* mode while retaining data and RTC functionality. Third, *e-Glass* includes two communication interfaces: 1) Bluetooth Low Energy (BLE) via the STMicroelectronics BlueNRG-MS chip and 2) USB 2.0. BLE enables data exchange with a proprietary app for internet connectivity and status monitoring. Since *e-Glass* processes data in real time, BLE throughput requirements are minimized, though it can stream data at higher energy costs–drawing 8.2 mA during transmission (0 dBm) and only 2.4 $\upmu$A in *Sleep* mode. Additionally, the system features a 64 Mbit external Flash memory for data logging, consuming 3.5 mA at peak during write cycles.

#### Electrical characterization

**Fig. 6:**
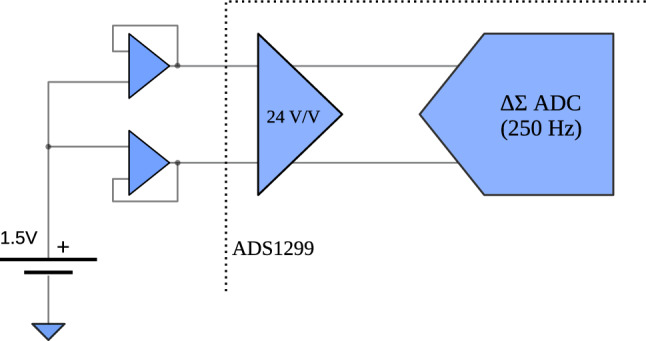
Input-referred noise measurement: external input short to measure the input-referred noise including all the external circuitry to ADS1299.

To validate the *e-Glass* noise performance, a minimum of 10,000 consecutive samples are acquired with the EEG channels short-circuited externally to the ADS1299 EEG front end (Fig. 6). As the ADS1299 is powered by a single supply, the channel inputs and reference are shorted to a known voltage within the power rail (i.e., 1.5 V from the battery). The root-mean-square (RMS) value of the IRN is estimated from the average power of the acquired samples, as defined in (1). The peak-to-peak value of the IRN is estimated by multiplying its RMS by 6.6^25^.

Once we obtained the IRN, the dynamic range (DR), noise-free bits (NFB), and effective number of bits (ENOB) are calculated using Equations (2),(3), and (4)^47^. Voltage followers at the active electrodes, input protection and filters have their noise contributions accounted for during measurement. The system is configured for maximum input gain (24 V/V) and lowest sampling frequency (250 Hz), aligning with its target operation.

Following a similar experimental approach, the system’s CMRR is evaluated by replacing the battery with a sinusoidal signal, generated using a LeCroy WaveStation (2012). CMRR is measured at 50 Hz by applying a sin wave of 100 $mV_P$, with an offset of 2.5 $V_{DC}$ as *e-Glass*’ ADS1299 is powered by a single supply. To guarantee correct input amplitude ($V_{IN_{P}}$), we used a Tektronix TDS 2024B oscilloscope to calibrate the testing signal. At least 2 minutes of data are used for the IRN estimation using the Discrete Fourier Transform (DFT). The CMRR is then calculated using (5):

1 $$\begin{aligned} IRN= & \sqrt{\frac{\sum _{j=1}^{n} v_n^2}{n}} \end{aligned}$$

2 $$\begin{aligned} DR= & 20 \cdot \log _{10}{\frac{VREF}{\sqrt{2} \cdot Gain \cdot IRN_{RMS}}} \end{aligned}$$

3 $$\begin{aligned} NFB= & \log _{2}{\frac{VREF}{\sqrt{2} \cdot Gain \cdot IRN_{pp}}} \end{aligned}$$

4 $$\begin{aligned} ENOB= & \log _{2}{\frac{VREF}{\sqrt{2} \cdot Gain \cdot V_{RMS}}} \end{aligned}$$

5 $$\begin{aligned} CMRR= & 20 \cdot \log _{10}{\frac{ V_{OUT_{P}} }{ V_{IN_{P}} } } \end{aligned}$$

#### EEG acquisition and processing

**Fig. 7:**
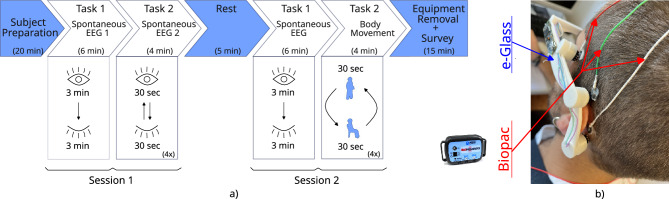
EEG acquisition: (**a**) experimental setup for acquiring data with *e-Glass* and Biopac simultaneously and (**b**) an example of how both systems are placed at a subject’s scalp, in which we see *e-Glass* along with BIOPAC’s electrodes.

Twelve healthy volunteers participated in an EEG acquisition study using *e-Glass* alongside a Biopac BN-EEG2 module (Biopac Systems, Inc). The study was divided into two experimental sessions, as presented in Fig. 7a, lasting for approximately one hour. Each volunteer undertook two data acquisition sessions of 10 minutes each, separated by a resting time of 5 minutes. Each session presented two tasks: 1) spontaneous EEG acquisition, including eyes open/closed changes, and 2) subject movement (i.e., sitting/standing) to elicit movement artifacts. The experiments followed the ethical principles of the Declaration of Helsinki and were approved by *La commission cantonale d’éthique de la recherche sur l’être humain* (CER-VD), Vaud - CH, under project ID 2022-01338. Furthermore, as per^48^, this sample size is considered appropriate for a pilot study assessing *e-Glass* reliability in EEG acquisition.

On the day of the experiment, each volunteer received a briefing on the experimental steps and signed an informed consent form. We then prepared each volunteer for the data acquisition by placing both the *e-Glass* system and the Biopac BN-EEG2, as shown in Fig. 7b. BN-EEG2 uses commercial Ag/AgCl EEG cup electrodes with EEG paste, while *e-Glass* electrodes were cleaned with alcohol to remove fat. Both systems’ electrodes were placed side by side for simultaneous signal acquisition. Since BN-EEG2 has only two EEG channels, we recorded *e-Glass*’ F7 and T7 signals for comparison. A synchronization trigger was coupled to both systems to allow posterior off-line data processing synchronization. In the case of *e-Glass*, the trigger signal was acquired using one of its analog inputs. An optocoupler was used to isolate the trigger circuit, avoiding unwanted conducted-noise coupling to *e-Glass* acquired signals.

The acquired signals are filtered between 1 and 30 Hz using a fourth-order Butterworth filter. Then, we divide the acquired data per experimental task using the trigger signal for synchronization. The spontaneous EEG is visually inspected for artifacts and used for validating *e-Glass*’s acquisition. First, we assess *e-Glass*’ data quality in the time domain by using Pearson’s correlation, which requires a sampling-frequency correction using the DTW method. The correlation is calculated over a 60-second window obtained from the Session 1 eyes open/closed task 1. Second, we apply Welch’s Periodogram to estimate the PSD from the 3-minute spontaneous EEG recorded during the eyes-closed phase of Session 1 (Task 1). The PSD is estimated considering 4-second EEG epochs. The results are plotted for visual inspection. In addition, we use the Bland-Altman method to assess spectral differences between equipment measurements^42^.

### e-Glass applications

Long-term EEG monitoring has the potential to provide key clinical information for personalized epilepsy treatment^43^ and can provide important markers to estimate cognitive workload (CW)^49^. Hence, in the following paragraph, we present methods used to evaluate the EEG-based epilepsy detection and CWM applications envisioned for *e-Glass* in^4,7,23^. For epilepsy detection, we report sensitivity (Sens), precision (Prec), F1-score, and FAR/day, as the data is very imbalanced. In addition to sensitivity, for CWM, we report specificity (Spec), accuracy (Acc), and Gmean of sensitivity and specificity as the data is balanced in this case. These scores are defined as follows:

6 $$\begin{aligned} & Sens = \frac{Tp}{Tp+Fn}, \qquad Prec = \frac{Tp}{Tp+Fp}, \qquad F1 = {\frac{2 \cdot Prec \cdot Sens}{Prec+Sens}}, \qquad FAR = \frac{Fp*24}{total\_time} \end{aligned}$$

7 $$\begin{aligned} & Spec = \frac{Tn}{Tn+Fp}, \qquad Acc = \frac{Tp + Tn}{Tp+Tn+Fp+Fn}, \qquad Gmean = \sqrt{Sens \cdot Prec} \end{aligned}$$

where Tp, Tn, Fp, and Fn stand for true positives, true negatives, false positives, and false negatives, respectively. $total\_time$ corresponds to the total amount of hours of data in the test set. All ML training/testing is done offline in both applications, using MATLAB and python routines.

Furthermore, we employ tools for code execution profiling and energy consumption estimation in order to assess memory requirements and battery lifetime in both applications. First, we use the RTOS tracing utility to account for thread execution time. Second, we combine the use of FreeRTOS tools with a manual account of dynamically allocated memory to estimate RAM. Last, we use the Otii Arc Pro^50^ commercial acquisition system to measure the average current consumption of *e-Glass* while executing the target ML-based application.

#### Epilepsy detection

We employ the CHB-MIT Scalp^15^ dataset to develop and evaluate an ML methodology for seizure detection. This dataset contains scalp EEG recordings from 24 pediatric patients, acquired using a bipolar montage at a sampling rate of 256 Hz. It includes a total of 198 annotated seizure events across 982.9 hours of data.

This application is evaluated in two scenarios: (1) using the data of all electrodes available for all subjects (i.e., 18 channels) and (2) using only the *e-Glass* channels (i.e., *F*7*T*7 and *F*8*T*8). We pre-process the EEG signals using a zero-phase, 4^th^-order Butterworth band-pass filter between [1, 20] Hz. The filtered data is divided into windows of $4\,s$ with a $0.5\,s$ step (87.5$\%$ overlapping) before extracting 56 classical literature features per EEG window, per EEG channel, as described in^7^.

For model evaluation, we employ a TSCV approach^7^, ensuring that all available data is used in the training and testing tasks. To ensure sufficient training data, we require a minimum of 5 hours of EEG recordings for the first model in the TSCV procedure, with at least one seizure event included. We post-process the results using the Bayesian approach described in^7^ before evaluating performance using the SZcore framework^51^, providing seizure detection metrics at the event level.

#### Cognitive workload monitoring

CWM can enhance human-machine interaction (HMI) by supporting task execution assistance, considering the operator’s cognitive state. To assess CWM solution tailored for *e-Glass*, we employ the in-house dataset described in^46^, which contains data of 24 volunteers (27.7 ± 4.8 years old) acquired while executing a simulated search and rescue (SAR) mission. Once more, the application is evaluated in two scenarios: (1) using the data of all electrodes available for all subjects and (2) using only the *e-Glass* channels (i.e., *F*7, *T*7, *F*8, and *T*8). The experimental setup is described in detail in^23^. In this case, besides bandpass filtering the data, we also employ the artifact removal technique developed for *e-Glass* system. We evaluate various data processing optimizations and achieve the best results considering: (1) EEG data windows of 56 s with 60% overlap, (2) a 200-tree RF model, and (3) only 18 features after running a 30-fold recursive feature elimination on cross-validation (RFECV).

## Conclusion

Real-time brain activity monitoring using wearable technologies enables continuous and non-invasive tracking of EEG in everyday environments on a personalized and long-term basis, supporting a wide range of health applications, from epileptic seizure detection to cognitive workload monitoring.

In this article, we present a state-of-the-art smart wearable system for unobtrusive EEG acquisition and processing in real-time, namely *e-Glass*, targeting personalized monitoring of neurological disorders. *e-Glass* tests shows it adhere to the guidelines for digital recording of clinical EEG of the IFCN^24^, have presented 1.07 $\mu$ $V_P$ of IRN, 18.41 noise-free bits (NFB), hight input impedance and CMRR. Moreover, in a pilot study in healthy subjects, *e-Glass*’ acquired data showed high correlation with the data from a research-grade EEG system (up to 0.93 in average Pearson’s correlation). We assessed two possible applications for *e-Glass*, the seizure detection and cognitive workload monitoring problem. The epilepsy data processing framework we tailored for ML-based EEG processing in resource-scarce devices as *e-Glass* reached 100% sensitivity in 11 out of 24 subjects, with average sensitivity and FAR of 63.8% and 2.35/day considering only two bipolar EEG channels (i.e., F7T7, F8T8). *e-Glass* can run the seizure detection ML algorithm for up to 28.5 hours on a single battery charge, which is on par with the desire of PWE for these type of devices.

On a more versatile application, we demonstrated that *e-Glass* may also be used for real-time CWM, having reached an accuracy of 74.5% and a 74.0% geometric mean of sensitivity and specificity on unseen data when validated on a in-house dataset. Thus, *e-Glass* may be used to enhance HMI by bringing the operator’s cognitive state information into consideration during work hours. Overall, *e-Glass* offers a state-of-the-art wearable platform for real-time EEG monitoring based applications as epilepsy detection and CWM.

## Data Availability

The seizure data used in this work is from a public database (CHB-MIT scalp EEG database), which could be accessed and downloaded via https://physionet.org/content/chbmit/1.0.0/. The dataset used for CWM was generated by Dr. Fabio Dell’Agnola, Dr. Ping-Keng Jao, Dr. Ricardo Chavarriaga, and Prof. José del R. Millán on the project no. PB2017-00295 (Swiss CER-VD ethical committee), and could be available through Prof. Millán’s authorization under reasonable request. Similarly, the e-Glass acquired data could also be available under reasonable request to the work’s corresponding author.
